# Relationship between subjective sleep disturbances and glycaemia in Chinese adults with type 2 diabetes: findings from a 1.5-year follow-up study

**DOI:** 10.1038/s41598-019-50814-9

**Published:** 2019-10-03

**Authors:** Chunrong Xu, Pan Zhang, Quanyong Xiang, Guiqiu Chang, Ming Zhang, Lei Zhang, Ting Li, Cheng Qiao, Yu Qin, Peian Lou

**Affiliations:** 1 Department of Endocrinology, Xuzhou Third People’s Hospital, 131 Huancheng Road in Xuzhou City of Jiangsu Province of People’s Republic of China, Xuzhou, 221004 China; 2 Department of Control and Prevention of Chronic Non-communicable Diseases, Xuzhou Center for Disease Control and Prevention, 142 West Erhuan Road in Xuzhou City of Jiangsu Province of People’s Republic of China, Xuzhou, 221006 China; 3 Department of Non-communicable Disease Control, Jiangsu Provincial Center for Disease Control and Prevention, 172 Jiangsu Road in Nanjing City of Jiangsu Province of People’s Republic of China, Nanjing, 210009 China; 4 Department of Control and Prevention of Chronic Non-communicable Diseases Xuzhou Center for Disease Control and Prevention, School of Public Health, Xuzhou Medical University, Xuzhou, China

**Keywords:** Type 2 diabetes, Type 2 diabetes

## Abstract

We wanted to determine whether subjective sleep disturbance was associated with serum glycated hemoglobin (HbA1c) in people with type 2 diabetes mellitus. In total, 944 randomly-selected people with diabetes completed the Chinese version of the Pittsburgh Sleep Quality Index (PSQI). Participants’ glycaemia was assessed using HbA1c in March 2016 and September 2017. The PSQI score and the change in score(△PSQI), and the HbA1c and its change (△HbAlc) were analysed by sex and age (30–45, 46–60, 61–75, and 76–89 years). Associations between time point PSQI and △PSQI with static HbA1c and △HbA1c were analysed using multiple linear regression. The results showed subjective sleep disturbance among people with diabetes was not correlated with serum HbAlc (β coefficient = 0.032, P = 0.103). However, cross-sectional multiple linear regression showed the relationship was present in women (β coefficient = 0.163, P < 0.01). In multiple linear regression, △PSQI score was correlated with △HbAlc value (β coefficient = 0.142, P < 0.01). The regression coefficient (β) for the relationship between △PSQI score and △HbA1c in men was greater than that in women, and for age was β_61–75years_ < β_46–60years_ < β_30–45years_. The strongest relationship between △PSQI and △HbA1c was in men aged 30–45 years (β = 0.452, P < 0.01). Subjective sleep disturbance among people with diabetes was not related to glycaemic status in the whole sample, but there was a correlation in women. The change in subjective sleep disturbance correlated with the change in glycaemia, most strongly in younger participants, especially men aged 30–45 years.

## Introduction

Traditional risk factors for diabetes are highly prevalent globally, including obesity, physical inactivity and energy-dense diets. Sleep disturbance, which is common in the general population, has also been shown to be a risk factor for diabetes, although it is less well-known. The ubiquity of traditional risk factors together with sleep disturbance has resulted in an unprecedented increase in the number of people with type 2 diabetes, with the number of people with diabetes globally estimated to reach 642 million by 2040^1^. The rate of increase in the prevalence of diabetes in China has also been marked, with an increase from 0.9% in 1980 to 10.9% in 2013^2^, This means that an estimated one in 10 adults in China has diabetes and the number of people with diabetes overall exceeds 100 million, which is more than in any other country^2^.

Subjective sleep disturbances have been reported in more than one-third of people with type 2 diabetes, which may be attributable to fear of poor blood glucose control and diabetic complications^3^. However, the relationship between subjective sleep disturbances and blood glucose levels was inconsistent in previous studies. Some studies have reported an inverse association between subjective sleep disturbances and poor glycemic control in people with type 2 diabetes^4–9^, whereas others reported no relationship between subjective sleep disturbances and serum hemoglobin A1c (HbA1c) level as an indicator of glucose status^10–13^. However, studies that report an association with HbA1c level did not fully exclude or adjust for important risk factors associated with poor sleep quality^4–9^, or did not include representative samples^7^. Therefore, the associations between subjective sleep quality and HbA1c shown in those studies were not robust.

Some previous studies used the presence of sleep disorders in their assessments^5^, which differ from subjective sleep quality^14^. In addition, sleep quality often changes. Most studies that examined the association between the subjective sleep quality and glycaemia were cross-sectional and only considered single time points, thereby ignoring the changes that may occur during the course of the disease. Furthermore, the few available cohort studies also had limitations, such as focusing on single time-points, not considering dynamic effects and often regarding HbA1c as the outcome variable.

Given the poorer glycaemic control and the huge burden of type 2 diabetes in China, an improved understanding of the relationships between subjective sleep disturbances and HbA1c level may provide important clues for improving glycaemic control in people with type 2 diabetes. It may also offer additional evidence that could be used to better evaluate these potential associations. Therefore, we conducted a prospective cohort study between March 2016 and September 2017 in Xuzhou city, China. We aimed to evaluate the relationship between subjective sleep disturbances over this period with HbA1c level in people with type 2 diabetes in a primary care setting in China.

## Methods

### Study design and participants

The diabetes registry system covers all communities in Xuzhou City, in northern Jiangsu province (eastern China). is moderately well-developed, and has a population of 10,000,000 across eleven regions, The probability proportional to size method was used to select the study sample in this area. First, according to the registered numbers of people with diabetes, five sub-districts(townships) were selected from each region with probability proportional to size. Within each sub-district, five neighbourhood communities or administrative villages were selected with probability proportional to size basied on the number of people with diabetes in each community. Finally, people with type 2 diabetes who met the inclusion criteria and were registered in health centers were selected using their medical records. At least 900 people were selected, assuming a prevalence of poor sleep quality of 30% in people with type 2 diabetes^3^, with 90% power and α = 0.05, and allowing for a drop-out rate of 10%. The baseline survey was performed in March 2016 and a final survey in September of 2017. We excluded people who had been diagnosed with type 2 diabetes in the preceding 6 months because they might not have achieved glycaemic stability.

The following exclusion criteria were also used in the study: (1) type 1 diabetes; (2) acute infection or other acute complication; (3) severe liver disease or kidney dysfunction; (4) severe heart failure (heart functional classes III and IV), severe cerebral vascular disease; (5) syndromes known to be associated with serious effects on sleep (e.g., severe diabetic peripheral neuropathy, retinopathy, somatic pain, pruritic skin disorders, nocturia, sleep apnea, and restless leg syndrome); (6) severe mental illness; (7) pregnancy or lactation; (8) other endocrine disorders, such as thyroid disease or chronic use of glucocorticoids; and (9) treatment with insulin, because exogenous insulin administration can increase sympathetic activity.

Written informed consent was obtained from all the participants. The study protocol was approved by the Xuzhou Center for Disease Control and Prevention (2011710). The procedures followed were in accordance with the standards of the ethics committee of Xuzhou Center for Disease Control and Prevention and with the Declaration of Helsinki (1975, revised 2013).

### Sleep quality

Sleep disturbance among people with diabetes is a complex symptom that includes impaired sleep quality and/or abnormal sleep duration^14^. We measured Sleep disturbance, which we defined as impaired sleep quality, using the Pittsburgh Sleep Quality Index (PSQI)^15^. The PSQI assesses sleep quality in the preceding month, and contains 19 entries on seven dimensions. Each dimension is scored from 0–3. The global PSQI score is thus 0–21; higher scores indicate worse sleep quality. In the Chinese version of the PSQI, a score >7 distinguishes poor sleepers from good sleepers. Accordingly, we defined a PSQI score > 7 as ‘poor sleep quality’. The change in PSQI score between visits was calculated by subtracting the baseline(2016) from the follow up (2017) PSQI score, and is presented as a △ value. A positive value implied sleep quality had deteriorated, a negative value implied it had improved and a zero value implied no change.

Because HbA1c is thought to reflect mean blood glucose over 3 months, we used this measurement as an index of glycaemic level in people with type 2 diabetes. Serum HbA1c was measured using high-performance liquid chromatography (Bio-Rad D-10 glycated hemoglobin meter). The change in HbA1c between visits was calculated by subtracting the baseline (2016) from the follow up (2017) HbA1c value, and is presented as a △ value. A positive value implied glycaemic level had deteriorated, a negative value implied glycaemic level had improved and a zero value implied no change over the study period.

### Other variables

Participants were interviewed privately face-to-face by trained interviewers using questionnaires which included age, gender, marital status, physical activity, net household income, level of education, cigarette smoking, alcohol consumption, time since diabetes diagnosis, number of comorbidities, number of diabetic complications, insulin use, depression, anxiety, and obstructive sleep apnea. The number of diabetic complications was determined by participants’ reported diagnoses of coronary artery disease, peripheral vascular disease, stroke, nephropathy, retinopathy, or neuropathy. Height (to the nearest 0.1 cm) and weight (to the nearest 0.1 kg) in light indoor clothing were measured at the clinic. Body mass index (BMI; in kg/m^2^) was calculated, and people were categorized as underweight (<18.5 kg/m^2^), normal weight (18.5−23.9 kg/m^2^), or overweight/obese (≥24.0 kg/m^2^). Depressive symptoms were scored using a nine-item patient health questionnaire (PHQ-9; total score range 0–27)^16^. Participants were defined as having moderate or severe depressive symptoms if they scored ≥10. Symptoms of anxiety during the preceding 2 weeks were recorded using the validated seven-item General Anxiety Disorder questionnaire (GAD-7; total score range 0–21)^17^. Participants were defined as having moderate or severe symptoms of anxiety if they scored ≥10. Obstructive sleep apnea was screened using the STOP-Bang questionnaire^18^, and a scored ≥3 were defined as obstructive sleep apnea. Pain was recorded using the method of Knutson *et al*.^4^. The question “how often have you had trouble sleeping because you had pain?” was used to identify people with sleep disturbance caused by pain; those that answered “3 or more times per week” were defined as suffering pain. Participants who had been diagnosed as having chronic pain were also included in this category. We excluded these individuals from our analyses of the association between sleep and glycemic control because chronic pain is likely to be a confounder.

### Statistical analysis

SPSS version 13.0 (SPSS, Chicago, USA) was used for all statistical analyses. Results are reported as the mean ± SD, or as numbers and percentages. Differences in continuous variables were evaluated using the Mann-Whitney U test and differences in categorical variables were assessed using Pearson’s χ^2^ test. One-way repeated measure ANOVA was used to explore HbA1c changes after 1.5 years by sleep quality. Bivariate spearman’s correlation was used to assess subjective sleep quality and HbA1c changes. Multivariate stepwise regression models was used for statistically significant variables in single factor analysis process. In addition, separate regression analyses were conducted by gender and age group, and the partial regression coefficient β were used to assess the impact of subjective sleep quality on HbA1c. P < 0.05 was considered to indicate statistical, no allowance was made for multiplicity.

## Results

### General characteristics of participants

**Figure 1:**
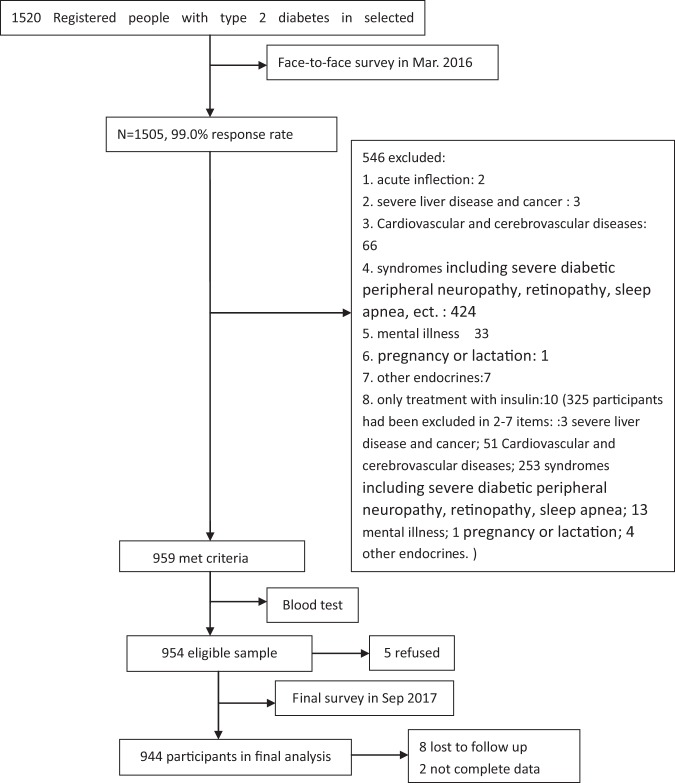
Data collection.

Figure 1 shows the data collection. Of the 1,520 individuals initially sampled, 566 (37%) participants who did not meet our study criteria were excluded, leaving 954 participants for inclusion in this study. 10 (1.0%) were lost to follow-up after 18 months, and 944 (64.3% women and 100% Han Chinese) adults with complete data were included in the analysis. Participants’ mean age was 64.13 ± 10.01 years (range, 30–89 years), and had been diagnosed with type 2 diabetes 6.65 ± 6.20 years before this study (range, 1–40 years). The mean HbA1c was 7.69 ± 1.46% and 35.91% (339/944) of the participants had an HbA1c of <7.0% (53 mmol/mol). The analysis showed that 17.48% were smokers, 12.92% were alcohol drinkers and 71.10% were being treated with oral hypoglycemic agents. The general characteristics of participants in the poor and good sleep quality groups are presented in Table 1.

**Table 1:** General baseline characteristics of participants with type 2 diabetes[N, %].

Variables		Sleep quality (Based on PSQI score)		P
		Good (≤7) (N = 633)	Poor (>7) (N = 311)	
Gender	Male	258 (40.8)	79 (25.4)	<0.001
	Female	375 (59.2)	232 (74.6)	
age groups	30–45	30 (4.7)	8 (2.6)	0.118
	46–60	193 (30.5)	80 (25.7)	
	61–75	333 (52.6)	185 (59.5)	
	76–89	77 (12.2)	38 (12.2)	
Age		62.29 ± 9.99	65.34 ± 9.47	0.006
Educational level	high school	108 (17.1)	30 (9.6)	<0.001
	Junior high school	203 (32.1)	72 (23.2)	
	Primary school	130 (20.5)	66 (21.2)	
	Illiteracy	192 (30.3)	143 (46.0)	
Drinking	Yes	96 (15.2)	26 (8.4)	0.005
	No	537 (84.8)	285 (91.6)	
Smoking	Yes	125 (19.7)	40 (12.9)	0.012
	No	508 (80.3)	271 (87.1)	
Family monthly income	≤3000¥	424 (67.0)	237 (76.2)	0.024
	3001–5000¥	116 (18.3)	44 (14.1)	
	5001–8000¥	70 (11.1)	25 (8.0)	
	≥8001¥	23 (3.6)	5 (1.6)	
BMI, mean (SD)		22.7 ± 5.6	23.9 ± 5.8	0.002
Time since diabetes diagnosis (years)		6.33 ± 6.12	7.32 ± 7.10	0.027
GAD-7	≥10 (Anxious)	31 (4.9)	38 (12.2)	<0.001
PHQ-9	≥10 (Depressed)	51 (8.1)	67 (21.5)	<0.001
HbA1c	<7%	246 (38.9)	93 (29.9)	0.007
Oral hypoglycemic agents		448 (70.8)	223 (71.7)	0.767

P values were obtained using the Fisher exact test for categorical variables and the Mann-Whitney U test for continuous variables. GAD-7 = Generalized Anxiety Disorder 7 scored on 0–21 scale, a low score.

favourable 10; PHQ-9 = Patient Health Questionnaire 9 scored on 0–27 scale, a low score.

favourable 10;. HbA1c = glycated hemoglobin.

### Impact of subjective sleep quality on HbAlc in people with type 2 diabetes at baseline

Among the 944 participants, the lowest PSQI score was 0, the highest was 18 and the mean was 6.43 ± 3.58. Overall, 32.94% (311/944) of participants had poor sleep quality; the mean PSQI scores were 10.79 ± 2.03 among poor sleepers and 4.29 ± 1.77 among good sleepers (t = 7.91, *P* < 0.01). Mean HbAlc values were 7.45 ± 1.50% among good sleepers and 8.17 ± 1.34% among poor sleepers (t = 10.43, *P* < 0.01).

### Effects of PSQI score on HbA1c at 18 months

Table 2 shows spearman’s correlations between PSQI score and HbA1c at 18 months. PSQI score correlated with HbA1c in those aged 61–75 and 76–89 years, but not in those aged 30–45 or 46–60 years. The relationship between the PSQI score and HbAlc were also assessed by gender and age (Table 2). PSQI score was correlated with HbAlc in women only, and in those aged 61–75 and 76–89 years, but not in those aged 30–45 or 46–60 years. PSQI score at single time points did not correlate with HbAlc in men overall, and when categorized by age, there was only a significant correlation in men aged 76–89 years. For women, PSQI score at 18 months significantly correlated with HbAlc in those aged 61–75 and 76–89 years.

**Table 2:** Relationships between subjective sleep status and HbAlc according to gender and age at baseline.

Age group	Male		spearman’s correlation coefficient	P	Female		spearman’s correlation coefficient	P	Total		spearman’s correlation coefficient	P
	Mean PSQI scores	Mean HbAlc value (%)			Mean PSQI scores	Mean HbAlc value (%)			Mean PSQI scores	Mean HbAlc value (%)		
30–45	5.28	8	−0.048	0.72	5.65	7.6	−0.131	0.66	5.47	7.8	0.012	0.98
46–60	5.64	7.7	0.011	0.93	6.36	7.7	0.114	0.15	6.12	7.7	0.129	0.34
61–75	5.74	7.8	0.176	0.17	7.16	7.1	0.373	<0.01	6.65	7.7	0.202	<0.01
76–89	5.72	7.5	0.186	<0.01	7.11	7.7	0.209	<0.01	6.57	7.6	0.187	<0.01
Total	5.67	7.7	0.035	0.68	6.85	7.7	0.322	<0.01	6.43	7.7	0.216	<0.01

P values were obtained using analysis of the spearman’s correlation analysis.

### Relationship between sleep quality and HbAlc in people with type 2 diabetes at 18 months

HbAlc value was set as the dependent variable and sleep quality as the independent variable in a linear regression equation, and the results of this analysis show that sleep quality correlated with HbAlc (Table 3 Model 1). When gender, age, educational level, family income, smoking, drinking, BMI, duration of diabetes, and anxiety and depressive symptoms were included as independent variables and the data were reanalysed using a multiple linear regression model, the result showed that sleep quality did not correlate with HbAlc (Table 3 Model 2). However, when the data for each gender were analysed separately there was a significant correlation between these variables in women (Table 3).

**Table 3:** Relationships between sleep quality and HbAlc in regression models at baseline.

	model 1	model 2	Male	Female
β coefficient	0.101	0.032	0.014	0.163
Standard error	0.049	0.011	0.009	0.010
Standardized β coefficient	0.182	0.135	0.114	0.211
P value	<0.001	0.103	0.283	<0.001

Model 1 was the unadjusted model.

Model 2 adjusted for gender, age, educational level, family income, smoking, drinking, BMI, duration of diabetes, and the presence of anxiety and depressive symptoms.

**Male and Female:** adjusted for age, educational level, family income, smoking, drinking, BMI, duration of diabetes, and the presence of anxiety and depressive symptoms.

### Impact of change in sleep quality on change in HbAlc in peoples with type 2 diabetes

To assess changes in HbA1c, we used a repeated measure ANOVA with participants grouped by sleep quality at 1.5 years. There was an interaction between sleep quality and time (*F* = 18.64, *P* < 0.01). PSQI score and HbAlc value increased over the 1.5 years between time points. Table 4 shows that the change in PSQI score was greater in men than in women (overall change 0.35, men 0.37, and women 0.34). The HbAlc increases were 0.19 overall, 0.20 in men, and 0.17 in women. The change in subjective sleep quality was correlated with the change in HbAlc (*P* < 0.01). Analysis of the relationship between △PSQI score and △HbAlc value by gender (Table 4) showed △PSQI score was correlated with △HbAlc value in both men and women. Analysis of relationship between △PSQI score and △HbAlc value by age (Table 4) showed a statistically significant correlation among 30–75 years. Evaluation of the relationship between △PSQI score and △HbAlc value by gender and age (Table 4) showed significant correlations among men aged 30–60 years and and women aged 61–75 years.

**Table 4:** Impact of changes from baseline to month 18 in sleep quality on HbAlc according to gender and age.

Age group	Male		spearman’s correlation coefficient	P	Female		spearman’s correlation coefficient	P	Total		spearman’s correlation coefficient	P
	ΔPSQI scores	ΔHbAlc value			ΔPSQI scores	ΔHbAlc value			ΔPSQI scores	ΔHbAlc value		
30–45	0.67	0.35	0.634	<0.001	0.58	0.31	0.536	<0.001	0.61	0.32	0.644	<0.001
46–60	0.48	0.29	0.648	<0.001	0.41	0.24	0.639	<0.001	0.43	0.26	0.609	<0.001
61–75	0.25	0.10	0.610	0.003	0.19	0.13	0.425	0.04	0.21	0.12	0.488	0.008
76–89	0.08	0.03	0.124	0.120	0.05	0.07	0.155	0.10	0.06	0.04	0.167	0.10
Total	0.37	0.20	0.625	<0.001	0.34	0.17	0.598	<0.001	0.35	0.19	0.599	<0.001

P values were obtained using analysis of the spearman’s correlation analysis.

### Relationship between change in sleep quality and change in HbAlc in people with type 2 diabetes

The △HbAlc value was set as the dependent variable and △PSQI score as the independent variable in a linear regression analysis. This showed that △PSQI score correlated with △HbAlc. When gender, age, educational level, family income, smoking, drinking, BMI, duration of diabetes, and anxiety and depressive symptoms were included as independent variables and reanalysed using a multiple linear regression model, △PSQI score was still correlated with △HbAlc (Table 5 Model 1).

**Table 5:** Relationships between change in sleep quality and change in HbAlc in regression models.

	β coefficient	Standard error	Standardized β coefficient	P value
Model 1	0.142	0.068	0.214	<0.001
Male	0.153	0.077	0.235	<0.001
Female	0.126	0.054	0.198	<0.008
**Age group**				
30–45	0.193	0.110	0.380	<0.001
46–60	0.142	0.068	0.214	<0.001
61–75	0.121	0.075	0.169	0.006
76–89	0.102	0.059	0.126	0.093
**Model Male**				
30–45	0.452	0.214	0.786	<0.001
46–60	0.174	0.007	0.201	<0.001
61–75	0.131	0.082	0.195	<0.001
76–89	0.116	0.075	0.138	0.062
**Model Female**				
30–45	0.382	0. 194	0.673	<0.001
46–60	0.127	0.085	0.323	<0.001
61–75	0.090	0.009	0.147	0.009
76–89	0.078	0.007	0.115	0.123

**Model I** = △PSQI score *vs* △HbAlc value, adjusted for age, educational level, family income, smoking, drinking, BMI, duration of diabetes, and presence of anxiety and depressive symptoms; Age group: adjusted for gender, educational level, family income, smoking, drinking, BMI, duration of diabetes, and presence of anxiety and depressive symptoms; **Model Male = **△PSQI score *vs* △HbAlc value in each age group of male adjusted for educational level, family income, smoking, drinking, BMI, duration of diabetes, and presence of anxiety and depressive symptoms; **Model Female** = △PSQI score *vs* △HbAlc value in each age group of female, adjusted for educational level, family income, smoking, drinking, BMI, duration of diabetes, and presence of anxiety and depressive symptoms.

1520 Registered people with type 2 diabetes in selected communities.

Finally, the relationship between △PSQI score and △HbAlc was analysed by gender. In adjusted models, the △PSQI score was correlated with △HbAlc value in both men and women (Table 5: women and men), but the correlation coefficient (β) for the relationship between △PSQI score and △HbA1c was greater in men than in women. Analysis of the association between △PSQI score and △HbAlc value by age showed a statistically significant correlation in the 30–75 years (Table 5: age group). The strength of the correlation between △PSQI score and △HbA1c level was β_61–75yeas_ < β_46–60years_ < β_30–45years_. The relationship between △PSQI score and △HbAlc was also evaluated using both gender and age (Table 5: Model 2 and Model 3). There was only a significant correlation in men aged 30–75 years and in women aged 30–60 years. The strongest relationship between △PSQI score and △HbA1c was in men aged 30–45 years.

## Discussion

This is the first study to evaluate the relationship between changes in sleep quality and in HbA1c in mainland China. There were three principal findings. First, subjective sleep quality did not correlate with HbAlc at 18 months across all participants. However, multivariable linear regression showed there was a correlation in women and a closer relationship between these measurements in older participants compared with younger participants. Second, △PSQI score was correlated with △HbAlc, with the correlation coefficient greater in men than t in women. Third, sleep quality and HbAlc value showed a greater increase over 1.5 years in younger participants compared with older participants, and the correlation between the two changes was strongest in aged 30–45 years.

The present study did not show that subjective sleep disturbance correlated with glycaemia at single time points in people with type 2 diabetes, which was not consistent with previous studies^4–9^. However, it is likely that the relationship between subjective sleep disturbances and glycaemia was confounded by other factors affecting sleep quality that were not entirely excluded in these previous studies^4–9^. In addition, the most recent study to report an association between poor sleep quality and poor glycemic control (odds ratio 0.82, 95% confidence interval: 0.74–0.89; *P* < 0.001)^19^ was compromised by the inclusion of medical inpatients who were >50 years, the fact that sleep quality was not analysed using a questionnaire, and that other problems affecting sleep quality were not considered. Therefore, we concluded that the findings of previous studies might have been confounded by other factors affecting the sleep quality of people with type 2 diabetes.

Consistent with previous studies^10–13^, we found that subjective sleep quality was not associated with the level of glycaemia. However, in previous studies, factors such as the use of insulin therapy^10–13^, and the presence of obstructive sleep apnea syndrome^10–13^, peripheral neuropathic pain^10–13^, or a depressive state^11–13^, were not fully considered. Therefore, we contend that our findings regarding the relationship between subjective sleep quality and glycaemic level are more reliable than those of previous studies.

The study showed that the association between subjective poor sleep quality and HbA1c is only present in women after adjusting for educational level, family income, smoking, drinking, BMI, duration of diabetes, and the presence of anxiety or depressive symptoms at baseline. The reasons for this finding may be as follows. First, some studies have suggested that sex and sex steroids influence sleep behavior and the development of sleep disorders^20,21^, and sleep disorders influence blood glucose status^5^. Second, depression in Chinese people is less easily identified than in the Western populations, because Chinese people tend to expressed depression as anxiousness, headaches, insomnia, chest discomfort, and dizziness, instead of “depression”^22^, Furthermore, women more likely to become depressed^22^, Chinese women are less educated than Chinese men, which may lead to a greater inability to express depression properly. Therefore, depressive symptoms might have been more likely to have been missed among women in China. People with diabetes who were depressed are less likely to have lower HbA1c levels than those who are not^23^. Although there was more female participants in current study, sleep quality was still related to glycemic level after adjusting for gender and other factors,which suggested that sleep quality was independently related to glycemic level.

We found that the correlation between subjective sleep quality and blood sugar was stronger in older than in younger people, especially in older women at baseline. A reason for this may be that despite older people being more likely to be taking antidiabetic medication than younger people, there was no difference in the level of glycaemic control between older and younger people^2^. Poor glycaemic control is characterized by hyperglycemia, which may result in more subjective sleep disturbances. Another reason may be that older women report the poorest sleep quality of any sex and age group^20^. Nevertheless, the relationship between subjective sleep quality and blood glucose was of most relevance in older women.

Our findings showed that changes in sleep quality are most likely to affect glycaemia in younger people with diabetes, especially young men. There are several possible reasons for this. First, younger people with type 2 diabetes in China tend to be less likely to be treated. In addition, diabetes treatment rates are lower for men than women^2^, which would be expected to lead to poorer glycaemic control, and in turn might have greater effects on the quantity and quality of sleep in men with diabetes^24^. Second, younger adults are more likely to work long hours or at night than older people, which disrupts their circadian rhythm and can lead to sleep disturbances^25^. Greater light exposure from computers and smart phones, which are widely used by younger adults in China, may also be associated with a higher risk of sleep disturbances^26^. Previous studies have suggested that sleep disturbance leads to a greater maladaptation in lifestyle in men than women, and poor lifestyle can directly or indirectly cause β-cell damage, leading to hyperglycemia^27^. Third, the widespread use of computers and smart phones leads to more inactivity and thus lower energy expenditure^28^. Sleep disturbances also have a negative impact on physical activity and eating behaviors^29^. Considering these factors together, people with a sedentary lifestyle and sleep disturbance exhibit aberrant blood glucose regulation and chronic hyperglycemia^27,30^. In addition, young men in China face social pressures such as work, marriage and family responsibilities, which influence sleep quality. Therefore, young men with type 2 diabetes showed greater changes sleep quality than their older counterparts.

Our findings suggest that changes in subjective sleep quality are associated with changes in glycaemia in people with type 2 diabetes, even in the absence of known factors influencing sleep quality. The relevance of subjective sleep disturbances to glycaemic level and possible strategies to improve sleep quality in type 2 diabetes merit further investigation, especially as sleep care is not routinely provided in community health centers in China. Our results thus have important implications for the understanding of the pathophysiology and management of poor sleep quality.

The strength of this cohort study was that it represented the first occasion on which the relationship between changes in subjective sleep quality in HbA1c had been evaluated using a validated questionnaire and a random heterogeneous sample of patients in a community-based setting in China. Furthermore, the effects of known factors affecting sleep quality in people with type 2 diabetes were excluded. Our findings provide strong evidence of a relationship between subjective sleep disturbance and glycemic level in people with type 2 diabetes in China. However, there were several limitations. First, we evaluated behavioral habits and disease using self-report questionnaires symptoms(including the PSQI). Although the questionnaires used have been shown to be valid and reliable, they were not objective measurements and the outcomes could have been affected by social desirability and recall bias. Second, anxiety and depressive symptoms were only assessed at baseline; therefore, we cannot rule out the development of symptoms of anxiety and depression during the period between assessments that may have affected glycaemia. Third, it is possible that some participants were misclassified, because self-report questionnaires were used to assess anxiety, depression, and poor sleep quality. Fourth, dietary intake, which impacts glycaemia and sleep quality, was not assessed. Fifth, this study included participants in a single region of China and more female, and it therefore should be carefully repeated in other areas and ethnic populations. Sixth, sleep quality were only assessed at baseline; therefore, we cannot rule out the development of poor sleep quality during the period between assessments that may have affected glycaemia.Seventh, this study included more female participant, and it therefore should be be avoided in other similar studies. Finally, the resultant connotation of youth must be careful explanation on account of a small number persons in this group.

We showed that subjective sleep quality is not correlate with HbAlc in a cross-sectional assessment of people with type 2 diabetes, although multiple linear regression analysis identified a correlation in women alone. However, sleep disturbance increased alongside an increase in blood glucose, with the correlation in men being stronger than in women. The changes in sleep quality and HbAlc were greater in younger participants than older participants, and the correlation was strongest in men aged 30–45 years. To future investigate this relationship between change in subjec**t**ive sleep quality and change in HbAlc, future studies should evaluate whether an improvement in sleep quality can reduce HbAlc in people with type 2 diabetes in China.

### Statement of assistance

The authors thank all the participants involved in the survey. The help of the District/County Centers for Disease Control and Prevention and clinics in Xuzhou City in the field surveys and data collection was very much appreciated. We thank Mark Cleasby, PhD, from Liwen Bianji, Edanz Group China (www.liwenbianji.cn/ac), for editing the English text of a draft of this manuscript.
